# Incidence of Leber hereditary optic neuropathy in 2019 in Japan: a second nationwide questionnaire survey

**DOI:** 10.1186/s13023-022-02478-4

**Published:** 2022-08-20

**Authors:** Fumio Takano, Kaori Ueda, Daniel A. Godefrooij, Akiko Yamagami, Hiroto Ishikawa, Hideki Chuman, Hitoshi Ishikawa, Yasuhiro Ikeda, Taiji Sakamoto, Makoto Nakamura

**Affiliations:** 1 Division of Ophthalmology, Department of Surgery, Kobe University Graduate School of Medicine, 7-5-2, Kusunoki-cho, Chuo-ku, Kobe, 650-0017 Japan; 2 Department of Ophthalmology, University Medical Center Utrecht, Utrecht, The Netherlands; 3 Inoue Eye Hospital, Chiyoda, Tokyo Japan; 4 Department of Ophthalmology, Hyogo College of Medicine, Nishinomiya, Japan; 5 Department of Ophthalmology, University of Miyazaki, Miyazaki, Japan; 6 Department of Ophthalmology, Kitasato University School of Medicine, Sagamihara, Kanagawa Japan; 7 Department of Ophthalmology, Kagoshima University Graduate School of Medical and Dental Sciences, Kagoshima, Japan

**Keywords:** Leber hereditary optic neuropathy, Epidemiology, Annual incidence, Mitochondrial DNA, Intractable disease, Prevalence

## Abstract

**Background:**

Leber hereditary optic neuropathy (LHON) is an acute or subacute optic neuropathy that mainly affects young males. The first nationwide epidemiological survey of LHON was conducted in 2014 in Japan, and LHON was officially designated as a rare intractable disease by the Japanese government in 2015. We conducted a second survey of the annual incidence of LHON in 2019, and estimated the total number of patients with LHON in Japan.

**Results:**

A questionnaire was sent to 997 facilities accredited by the Japanese Ophthalmological Society and/or affiliated with the councilors of the Japanese Neuro-Ophthalmology Society. Responses were received from 791 facilities, with a response rate of 79%. Fifty-five newly diagnosed cases (49 males and 6 females) of LHON were reported from 35 institutions in 2019, with a median age of 28.5 for males and 49.5 years for females. The total number of newly diagnosed cases was calculated as 69 (62 were males and 7 were females, 95% confidence interval 55–83), and the total number of patients was estimated to be 2491 (95% confidence interval: 1996–2986), suggesting a prevalence of LHON in Japan of 1:50,000.

**Conclusion:**

The incidence of LHON in 2019 was lower than the estimate in 2014, whereas its prevalence may be similar to that reported in other countries. The accurate estimation of the incidence and prevalence of patients with LHON requires prospective registration.

## Background

Leber hereditary optic neuropathy (LHON) is a maternally inherited optic neuropathy, that causes acute or subacute optic neuropathy characterized by central scotoma and marked reduction of visual acuity [1]. Over 95% of patients have one of three mtDNA point mutations, known as primary point mutations. These primary point mutations cause a deficiency in electron transfer complex I that leads to the apoptotic cell death of retinal ganglion cells. However, the full pathophysiology of LHON is not yet fully understood. Unlike other mitochondrial diseases, LHON does not occur by mtDNA point mutations alone, and it is believed that some environmental and/or epigenetic factors are involved in the development of LHON [2]. LHON has been designated as a rare intractable disease by the Japanese government, following the establishment of designated criteria in 2015 [3].

The prevalence of LHON is reported to be about one in 31,000–520,000 [4]. In Japan, the incidence of LHON was not known until we conducted the first nationwide epidemiological survey in 2014. On the basis of this survey, we estimated the number of newly diagnosed patients with LHON as 117 (109 males and 8 females) with a 95% confidence interval of 81–153 [5].

Most of the affected individuals are male, both in Japan and worldwide [5–8]. However, the male to female proportion may be increasing for unknown reasons. The proportion of males was reported to be 68.1% [6] in 1973, but was 92.1% [7] in 1995 and 93.2% in our 2014 survey [5]. In addition, the proportion of male patients in one large LHON family was reported to increase over generations [8]. However, whether such a skewed sex proportion is maintained in Japan has yet to be confirmed.

Although most patients with LHON have one of the three major mutations, there have been an increasing number of reports of LHON with other rare mutations [9–11]. In the previous survey, we investigated only LHON cases with the three primary mutations, and thus whether and to what degree newly diagnosed Japanese patients with LHON harbor rare mutations has not yet been systematically evaluated.

The purpose of this study was to investigate the number of newly diagnosed patients with LHON with any causative mtDNA mutations in 2019, and use these data to estimate the annual number of newly diagnosed cases and the total number of patients with LHON in Japan. [12–14]

## Results

Of the 997 facilities to which we sent the questionaries, 791 responded, a response rate of 79%. Fifty-five cases (49 males and 6 females) were reported as newly diagnosed patients with LHON in 2019, as described in Table 1. The male to female ratio was 90%, comparable to the results of our previous study [5].

**Table 1:** Summary of the results of the survey

Results of the questionnaire	
Facilities to which questionnaires were sent	997
Facilities that responded (%)	791 (79%)
Facilities where patients were reported	35
1 Patient	26
2 Patients	5
3 Patients	2
5 Patients	1
8 Patients	1
Total patients reported	55
Male	49
Female	6
Number of patients with primary mutations	
11,778	45
14,484	8
3460	0
Number of patients with rare mutations	
11,696	1
11,281	1

The questionnaire surveyed the number of newly diagnosed cases of LHON in 2019, sex, and location of mtDNA mutation

**Fig. 1:**
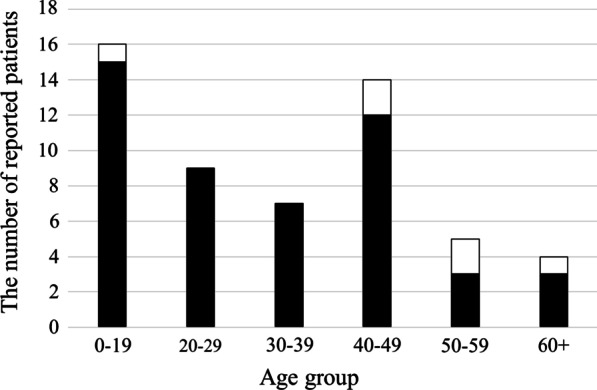
Histogram of the number of reported patients by age group. The black part of each bar shows the number of males, and the white part shows the number of females

Figure 1 shows the distribution of age at onset of the reported cases. The median (interquartile range) age was 31.0 (17.0–45.5) years for males and 49.5 (38.8–54.0) years for female patients. The youngest patient was 7 years old, and the oldest was 66.

With respect to the mtDNA mutations harbored by the patients, 45 individuals had the m.11778 G > A mutation, eight had the m.14484 T > C mutation, whereas none had the m.3460 G > A mutation. One occurrence of each of the m.11696 G > A and m.12811 T > C mutation was also reported. These mutations are described as rare mutation loci in the diagnostic criteria [3].

Based on the above response rate, the number of surveyed facilities, and the reported number of patients, the incidence number (*Ir*; the number of patients with newly diagnosed LHON in 2019) was calculated to be 69 (62 males and 7 females, 95% confidence interval [CI]: 55–83), while the *Tr* (the total number of patients with LHON in Japan) was calculated to be 2491 (2333 males and 158 females; 95% CI: 1996–2986). Given the total Japanese population in 2019 of 126,167,000 [15], the prevalence of LHON was estimated to be 1: 50,649 (= 126,167,000/2491).

## Discussion

This is the second nationwide epidemiological study of LHON in Japan. In questionnaire surveys, high response and sampling rates, as well as the avoidance of duplicate registrations, are the keys to increasing the reliability of the results.

The response rate improved significantly in this survey (79.3%) compared to the previous survey (61.6%). The major reason for this increase was an increased awareness of and interest in this project, because this was the second survey. The next most important factor was a change in the facilities to which the questionnaires were sent. In the previous survey, all facilities accredited by the Japanese Ophthalmological Association and all facilities with at least one member of the Japanese Neuro-Ophthalmology Society were surveyed, a total of 1397 facilities. This time, however, we reduced the number of facilities to which questionnaires were sent to 997. The questionnaire was sent to facilities accredited by the Japanese Ophthalmological Association, as in the previous survey, but the number of facilities affiliated with the Japanese Neuro-Ophthalmology Society was limited to those to which the council members belonged. This change was made because it was assumed that the council members of the Japanese Neuro-Ophthalmology Society would generally be more familiar with the diagnosis of LHON than the general membership, would be less likely to overlook or misdiagnose the condition, would be more likely to receive referrals of eligible candidates, and would be more likely to respond positively to the questionnaire.

In this study, the extraction rate, which refers to the proportion of the number of institutions we sent the questionnaire relative to the possible number of institution where diagnosis of LHON could be made, was assumed to be 1 for the analysis. The extraction rate will be less than 1 if a patient with LHON is seen at a facility other than the one included in this study and is not referred to the study facility. If this actually happened and the true extraction rate was low, the number of newly diagnosed cases could have been underestimated. In practice, the number of newly diagnosed cases reported in the questionnaire was lower in this survey than in the previous survey. However, this decrease was not due to a decrease in the extraction rate, but rather due to a decrease in the number of cases of duplicate registration. In the previous survey, there were several reports of patients who had developed the disease before the target year, and duplicate reports of the same patient from several institutions by multiple members of the Japanese Neuro-Ophthalmology Society. The previous survey was the first national epidemiological survey on LHON, and the requirement that the target population was limited to newly diagnosed patients in the past year may not have been fully understood by the surveyed institutions and researchers. In addition, the LHON accreditation criteria had only been established relatively recently, so the diagnosis may have been inaccurate in less experienced facilities. This time, the bias caused by duplicate registrations and inaccurately diagnosed cases was considered to be very low because, as mentioned above, the survey was limited to facilities with more experience and sufficient expertise, and because the understanding of the diagnostic criteria appears to have spread sufficiently, with the designation of LHON as an intractable disease. Since most LHON patients are school-aged to mature males, have subacute, binocular vision loss, and an uninsured mitochondrial genetic test is essential for diagnosis, it is highly likely that patients will visit a neuro-ophthalmologist, rather than being treated by general ophthalmologists, neurologists, or other institutions outside the scope of this study. Therefore, the extraction rate is expected to approximate to 1 with a fairly high probability. However, since the possibility of duplicate registrations cannot be completely ruled out, we may have overestimated the number of newly diagnosed patients.

In the current survey, the age distribution and sex ratio were almost the same as in the previous survey. Compared to previous reports from other countries, the average age of onset was higher, and males accounted for a higher percentage of cases. As in the previous survey, this increase in the age of onset is thought to be due to the increasing average life expectancy of Japanese people and the declining birth rate. As the decline in the birthrate and the aging of the population accelerates, the age of onset of LHON in the Japanese population is likely to continue to rise. The reason for the greater susceptibility of males is unknown. Although the number of cases was small, the age of onset of LHON was higher in women than in men. It has been suggested that sex hormones may play a role in the development of LHON [16]. Estrogen is presumed to act as a protective factor for retinal ganglion cells and other nerve cells [17], and may delay the onset of the disease in female mutation carriers until around menopause, when the amount of estrogen in the body decreases.

The other difference between this and the previous survey is the mtDNA mutation sites investigated. In the previous study, only the three primary mutations were included, but in this study, all mutations reported to be associated with LHON in the designation criteria, including rare mutations, were included. As a result, the present study reported the presence of patients with two mutations, m.12811 T > C and m.11696 G > A, which have been reported as rare mutations in LHON [9–11].

There are several limitations to this study. First, because it is a single-year questionnaire survey, it is affected by variations in patient population estimates due to differences in survey years. As noted above, the number of newly diagnosed cases was estimated to be lower than in the 2014 survey. Periodic epidemiological surveys and prospective all-patient registries should be conducted to obtain more accurate epidemiological trends.

Secondly, the number of patients in this study was calculated based on the assumption that the life expectancy of individuals with LHON is equivalent to that of healthy people. However, since there are reports of lower life expectancy in patients with LHON [18], the total number of patients calculated in this study may be an overestimate.

Third, as a method for estimating the total number of patients, we used the formula published by Godefrooij et al. to estimate the number of keratoconus patients in The Netherlands. As discussed by the authors, this method is only an estimation, producing a rough estimate of the prevalence, and it is still necessary to conduct a full patient registry to accurately determine the actual number.

## Conclusions

A second national epidemiological survey on LHON was conducted. As a result, the number of newly diagnosed patients with LHON in 2019 was estimated to be 69, and the total number of LHON patients in Japan was estimated to be 2400, with a prevalence similar to other countries. A prospective all-patient registry project is necessary to accurately determine the actual number of patients.

## Methods

### Questionnaire, diagnostic criteria, and participating facilities

We developed a questionnaire to estimate the number of patients with LHON developed in 2019. The questionnaire included the presence or absence of newly diagnosed cases of LHON in 2019, the age and sex of the individuals involved, and the nucleotide position of the mtDNA mutations. The diagnosis of LHON complied with the diagnostic criteria established in 2015, as published by the Research Committee on the Epidemiology of Intractable Diseases of Retinochoroidal and Optic Nerve Atrophy in conjunction with the Japanese Neuro-ophthalmological Society, and authorized by the Ministry of Health, Labour and Welfare in Japan [3].

We collected LHON cases diagnosed in 2019 as “newly diagnosed” cases in 2019, regardless of the period from onset to diagnosis. Since patients with LHON experience rapid binocular loss of vision, it was assumed that they would not be left unexamined, and would almost always seek medical attention. In addition to genetic testing, orbital MRI and fluorescein fundus angiography are necessary to diagnose LHON, so it was presumed that the primary medical institution first visited by the patient would almost always refer the patient to an institution specializing in neuro-ophthalmology. In Japan, such higher-order medical institutions are considered to be facilities accredited by the Japanese Ophthalmological Society, or facilities affiliated with the councilors of the Japanese Neuro-Ophthalmology Society. Therefore, it is reasonable to assume that by sending the questionnaire to these facilities (997 in total), we could identify almost all of the new patients with LHON in the survey year.

### Statistical analysis

The survey was based on the Nationwide Epidemiologic Survey Manual, published by the Research Committee on Epidemiology of Intractable Disease [12–14]. The incidence number (*Ir*) was calculated as in the manual and in our previous survey [5] as follows;

$${\text{Ir}} = \frac{{\Sigma {\text{iNi}}}}{{{\text{N}}/{\text{n}}}},$$

where *Ir* denotes the estimated total number of patients (as units of patients with newly diagnosed LHON), *i* is the actual number of patients reported in response to the questionnaire (*i* = 0, 1, 2…), *Ni* is the number of responding facilities with *i* patients, *N* is the total number of responding facilities, and *n* is the total number of facilities surveyed.

In previous similar epidemiological surveys of intractable diseases [12–14], the facilities were often selected randomly using stratified sampling from all of the facilities visited by a patient with the disease of interest. Thus, the number of facilities surveyed is usually much smaller than the total number of facilities when the corresponding facilities are classified into general hospitals or clinics with a small number of beds. In this situation, *n* is assumed to be much bigger than the number of facilities surveyed. However, we assumed that newly diagnosed LHON cases were referred almost immediately to facilities with affiliated specialists who had expertise in LHON for the following reasons. First, individuals with a recent onset of LHON notice a rapid and profound decline in their central vision in both eyes, but they rarely exhibit extraocular symptoms. Second, given the necessity for molecular diagnosis and the lack of response to steroid therapy, these patients are highly likely to be referred to tertiary or special institutes with affiliated neuro-ophthalmological specialists. The facilities to which we sent the questionnaire covered most of these specialists. Thus, all of the facilities were considered to be categorized as “selected departments” according to previous similar epidemiological surveys of intractable diseases [12–14]. Therefore, we did not consider that the facilities should be stratified based on the hospital type or the number of hospital beds, so *n* was equal to the number of surveyed facilities.

The 95% CI of *Ir* was *Ir* ± 1.96 × *s*, where *s* is the estimated standard error of *Ir*, computed as follows: [12–14]

$${\text{S}} = \sqrt {\frac{{\sum i^{2} \times \frac{{N_{i} }}{N} - \left( {\sum i \times \frac{{N_{i} }}{N}} \right)^{2} }}{n - 1} \times n^{3} \left( {\frac{1}{N} - \frac{1}{n}} \right)}$$

A patient reported by multiple facilities was treated as a “duplicate” according to the information obtained from the questionnaire and/or via direct contact with the physicians.

In this survey, we also calculated the estimated total number of LHON patients (*Tr*) based on the National Population Survey reported by the Ministry of Health, Labour, and Welfare [19] and the *Ir* calculated as described above, according to a previous report [20] as follows;

*Tr* = *Ir* × the amount of time that people are at risk × the fraction of life for which the average LHON patient is affected.

In this formula, the amount of time that people are at risk was approximated to be 60 years for a man and 50 years for a woman, because the questionnaire revealed that the onset of age ranged from 7 to 66 for male patients and from 17 to 66 for female patients. The fraction of life for which the average LHON patient is affected was calculated based on the average longevity and the average life expectancy at the year of median onset of LHON for each sex in 2019 [15]. The average longevity was 81.41 years for a man and 87.45 years for a woman in 2019, whereas the average life expectancy for a man aged 31 years, the median age at onset for male LHON patients, was 51.06 years and that for a woman aged 49 years was 39.44 years. The average male and female LHON patient are therefore affected with LHON for 62% (51.06/81.41 multiplied by 100) and 45% (39.44/87.45 multiplied by 100), respectively, of their lives. Thus, the obtained values of 0.62 and 0.45 were used as the fraction of life for which the average male and female LHON patient, respectively, is affected.

## Data Availability

All data generated or analyzed during this study are included in this manuscript and its additional files.
